# Chronic Musculoskeletal Pain Moderates the Association between Sleep Quality and Dorsostriatal-Sensorimotor Resting State Functional Connectivity in Community-Dwelling Older Adults

**DOI:** 10.1155/2022/4347759

**Published:** 2022-04-07

**Authors:** Soamy Montesino-Goicolea, Pedro A. Valdes-Hernandez, Yenisel Cruz-Almeida

**Affiliations:** ^1^Department of Community Dentistry and Behavioral Sciences, University of Florida, Gainesville, USA; ^2^Pain Research and Intervention Center of Excellence, University of Florida, Gainesville, USA; ^3^Evelyn F. and William L. McKnight Brain Institute, University of Florida, Gainesville, USA; ^4^Institute on Aging, University of Florida, Gainesville, USA; ^5^Center for Cognitive Aging and Memory, University of Florida, Gainesville, USA; ^6^Department of Neuroscience, College of Medicine, University of Florida, Gainesville, USA

## Abstract

*Aging* is associated with poor *sleep quality* and greater chronic pain prevalence, with age-related changes in brain function as potential underlying mechanisms. *Objective*. The following cross-sectional study aimed to determine whether self-reported chronic musculoskeletal pain in community-dwelling older adults moderates the association between sleep quality and resting state functional brain connectivity (rsFC). *Methods*. Community-dwelling older individuals (mean age = 73.29 years) part of the NEPAL study who completed the Pittsburg Sleep Quality Index (PSQI) and a rsFC scan were included (*n* = 48) in the present investigation. To that end, we tested the effect of chronic pain-by-PSQI interaction on rsFC among atlas-based brain regions-of-interest, controlling for age and sex. *Results and Discussion*. A significant network connecting the bilateral putamen and left caudate with bilateral precentral gyrus, postcentral gyrus, and juxtapositional lobule cortex, survived global multiple comparisons (FDR; *q* < 0.05) and threshold-free network-based-statistics. Greater PSQI scores were significantly associated with greater dorsostriatal-sensorimotor rsFC in the no-pain group, suggesting that a state of somatomotor hyperarousal may be associated with poorer sleep quality in this group. However, in the pain group, greater PSQI scores were associated with less dorsostriatal-sensorimotor rsFC, possibly due to a shift of striatal functions toward regulation sensorimotor aspects of the pain experience, and/or aberrant cortico-striatal loops in the presence of chronic pain. This preliminary investigation advances knowledge about the neurobiology underlying the associations between chronic pain and sleep in community-dwelling older adults that may contribute to the development of effective therapies to decrease disability in geriatric populations.

## 1. Introduction

Sleep is essential for restoring our bodies physiological processes, with lack of sleep negatively impacting cognitive and physical performance, and ultimately, quality of life. Sleep quality pronouncedly deteriorates with age, and approximately 50% of older adults usually complain of sleep disturbances. Similarly, chronic pain is highly prevalent in older individuals, often associated with lower cognitive and physical function. Evidence implicates sleep disturbance as an essential risk factor for increased pain, with existing findings suggesting that sleep disturbance predicts the onset or exacerbation of clinical pain [1–6]. Additionally, laboratory studies have demonstrated that experimental sleep disruption reduces pain inhibitory function in healthy people and those with chronic pain [7, 8]. However, there is little research examining the potential mechanisms at the intersection of these two common conditions in the older population.

While both sleep disturbances and chronic pain are associated with altered brain structure and function (see reviews by [9, 10]), aging is also known to change the brain. Indeed, the complex pain experience is sculpted by dynamic interactions in the brain, which may be further impacted by brain aging processes. Thus, there is a need to understand the brain mechanisms at the intersection of pain and sleep in the older population. Identifying common neurobiological mechanisms underlying age-related sleep problems and chronic pain can contribute to the development of effective therapies that would decrease the progression to disability in this vulnerable population.

Resting-state Magnetic Resonance Imaging (MRI) functional connectivity (rsFC), a technique particularly useful to examine the functioning of brain networks at rest, has recently provided preliminary evidence of the effects of poor sleep quality on brain networks during wakefulness [11–20]. Similarly, rsFC is altered in chronic pain states [21–23] including in older individuals [24, 25]. To our knowledge, no study to date has probed the rsFC for the interplay between sleep quality and chronic musculoskeletal pain in older adults. Therefore, the present exploratory study evaluates the associations of rsFC with the interaction of self-reported sleep quality, measured using the Pittsburg Sleep Quality Index (PSQI), and self-reported musculoskeletal pain in cognitively healthy older adults. We hypothesize that the association between PSQI scores with rsFC brain networks involved in sleep and pain perception will be dependent on the presence of chronic musculoskeletal pain in community-dwelling older adults.

## 2. Methods

### 2.1. Participants

This is an observational cross-sectional study that included community-dwelling older adults (over 60 years of age), native English speakers enrolled as part of the screening process for a larger NIH-funded study at the University of Florida (UF) studying pain, aging, and mobility function (Neuromodulatory Examination of Pain and Mobility Across the Lifespan [NEPAL]). The NEPAL study was powered (80% power, alpha = 0.05) to examine brain differences between older and younger adults, and the present study examines secondary outcomes collected from older participants. Participants were recruited through newspapers, ads, posted flyers, and word-of-mouth referrals at UF Health Sciences. The study and recruitment methods were not focused on recruiting individuals with a specific chronic pain condition. Study advertisements were targeted to study brain aging to avoid potential recruitment bias in relation to pain. Potential participants were screened over the phone and in person from September 2015 to January 2019. Exclusionary criteria included the following conditions: inability to consent, MRI ineligibility, serious psychiatric conditions (e.g., schizophrenia, major depression, bipolar disorder), history of alcohol/drug abuse (<1 year); Alzheimer's, Parkinson's, Epilepsy, and other known intra-cerebral pathology; significant cognitive impairment as evidenced by a score equal to or less than 77 on the Modified Mini-Mental State Examination (3 MS); hospitalizations for mental health reasons in the past year; chronic use of narcotic medications; serious systemic (uncontrolled diabetes self-reported HA1C >7), neurological, or cardiovascular disease (uncontrolled hypertension >155/90 mm Hg); systemic rheumatic disorders (i.e., rheumatoid arthritis, systemic lupus erythematosus, fibromyalgia); self-reported HIV or AIDS; and excessive anxiety regarding protocol procedures. In addition, participants did not report any sleep disorders, such as obstructive sleep apnea, narcolepsy, or insomnia. All participants provided informed consent prior to undergoing further screening and any of the experimental procedures, and the study was reviewed and approved by the UF Institutional Review Board (IRB).

### 2.2. Study Outcomes

#### 2.2.1. Demographics and Other Potential Confounding Variables

During a baseline visit, demographics and general health history information were obtained. In addition, the Montreal Cognitive Assessment (MoCA) was administered to the participants. MoCA score measures global cognitive abilities and function [26]. It ranges from zero to 30, with a score of 26 and higher generally considered normal global cognition. The Center for Epidemiologic Studies Depression Scale (CES-D) [27] was also administered to assess depressive symptoms experienced by participants during the last week on a 4-point Likert scale. In addition, Positive and Negative Affect Schedule (PANAS) [28] and the State-trait anxiety inventory (STAI) [29] was administered to measure positive and negative affect using 20 items on a 5-point Likert scale. The State-trait anxiety inventory (STAI) [29] consists of 20 items with response options based on a 4-point Likert scale (e.g., from “Not at all” to “Very much so”) to assess anxiety symptoms with higher scores indicating greater anxiety.

#### 2.2.2. Predictors of Interest

During the baseline visit, participants also provided *self-reported* measures of *pain and sleep quality*. To avoid bias in the data collection, participants were assigned to the chronic pain groups in a post-hoc fashion (i.e., after all data collection where individuals reporting pain on most days during the past 3 months were assigned to the chronic pain group, while those not meeting this criterion were classified as no-pain controls. This is the definition of chronic pain consistent with the Task Force for the Classification of Chronic Pain consensus for the 11th version of the International Classification of Diseases of the World Health Organization (WHO), and has been used to define participants with musculoskeletal pain in the literature [30–35], including neuroimaging studies [36–39]. Participants also completed a standardized pain history interview regarding the presence of pain across several body regions using a validated body manikin [40]. Participants were asked about the locations of their worst pain, its duration, as well as its frequency during the past week. The Western Ontario and McMaster Universities Osteoarthritis Index was also administered to assess global joint pain. At the end of this laboratory session, participants were asked to fill out the Pittsburgh Sleep Quality Index (PSQI) [41] to assess sleep quality. The PSQI is considered an accepted reference or gold standard for self-perceived sleep quality. It is an effective instrument used to measure the quality and patterns of sleep in older adults during the past month. It differentiates “poor” from “good” sleep by measuring seven domains: subjective sleep quality, sleep latency, sleep duration, habitual sleep efficiency, sleep disturbances, use of sleep medication, and daytime dysfunction over the last month. The participant self-rates each of these seven areas of sleep. Scoring of the answers is based on a 0 to 3 scale, whereby 3 reflects the negative extreme on the Likert Scale. A global sum of “5” or greater indicates a “poor” sleeper.

#### 2.2.3. Functional Connectivity Measure

In a separate visit within the next four weeks of the baseline visit, brain images were acquired at the University of Florida McKnight Institute with a 3T Philips Achieva MR scanner (Philips Medical Systems, Best, The Netherlands) using a 32-channel head coil. Before the session, participants completed a questionnaire rating their current clinical pain. Resting-state gradient-echo-planar imaging (EPI) data (fMRIs) were acquired with 38 Philips-interleaved slices, TR = 2 sec, TE = 30 msec, FOV = 224 × 224 × 133 mm, 64 × 64 × 38 mm matrix, flip angle = 90°, in plane resolution = 3.5 × 3.5 mm, slice thickness = 3.5 mm, 0 mm skip and SENSE factor = 2 in the AP direction. The run lasted 10 minutes, and 300 time points were acquired. Whole-brain high-resolution three-dimensional T1-weighted anatomical images were also acquired using an MP-RAGE sequence with sagittal plane, FOV = 240 mm × 240 mm × 170; 1 × 1 × 1 mm isotropic voxels, TR = 7.1 msec, TE = 3.2 msec and flip angle = 8 deg.

We preprocessed the functional MRIs using the standard SPM12 (https://www.fil.ion.ucl.ac.uk/spm) pipelines for slice timing and motion/unwarp correction. We used SPM12's unified segmentation [42] to segment time averaged fMRIs into gray/white matter and cerebrospinal fluid and spatially normalize them to the MNI space. Given the remaining large morphometric variability in the sample, we refined the normalization with SPM12's default DARTEL [43], to generate sample-specific template segmentations in the MNI space with a final resolution of 3 × 3 × 3 mm. Since DARTEL delivers large deformations, we used the *pushforward* warping method to preserve all the data from the native fMRIs [44]. We applied the same segmentation and DARTEL procedures independently to the T1-weighted images.

We calculated the average within Region-of-Interests (ROIs) of the warped fMRIs, excluding voxels outside the individual's gray matter mask, using CONN version 19a [45]. The ROIs were defined from the Harvard-Oxford AAL anatomical atlas [46], comprising a total of 132 cortical, subcortical, and cerebellar structures and the brainstem. This atlas can be found at https://fsl.fmrib.ox.ac.uk/fsl/fslwiki/Atlases. We temporally filtered the ROI time series using a band-pass filter between 0.008–0.09 Hz and denoised them using the General Linear Model (GLM). The noise regressors were (i) the six motion parameters and their temporal derivatives; (ii) the *scrubbing* penalizing artifactual time points, and the first five spatial principal components—*aCompCor* [47]—of the spatially non-smoothed preprocessed fMRIs within white matter and cerebrospinal fluid; and a 10 min duration boxcar function (*rest* regressor) convolved with the canonical hemodynamic response function (HRF), as well as its first and second temporal derivatives to account for departures from its canonical behavior. Quality control of the denoising was based on (i) visual comparison, before and after denoising, of the histograms of temporal correlation between random 1000 brain voxels of the preprocessed fMRIs and on (ii) visual inspection of the carpet plots of the BOLD signals in all voxels [48]. We evaluated departures from the null distribution, obtained with permutations of the correlation between the functional connectivity in random 1000-node networks and brain displacements and between the former and global signal changes [49]. No subject in our sample was discarded due to this quality control.

We converted the denoised ROI time series to percent signals and centered them to have zero mean. We calculated the *ROI-to-ROI rsFC* (R2R connectivity) between a pair of ROI time series as the zero-lagged bivariate weighted temporal correlation, Fisher-transformed to have a normal distribution.

### 2.3. Statistical Methods

Data was determined to be missing-at-random, and a listwise deletion was employed in all analyses. An independent sample *t*-test was used to test for differences in continuous variables between the pain and no-pain groups. The *χ*^2^ test was used to test for differences in categorical variables (e.g., sex, race, education level, marital status, and income) between the pain and no-pain groups. There were no significant differences between the groups regarding clinical and demographic characteristics (Table 1), except for total PSQI and the momentary pain intensity in the MRI.

#### 2.3.1. Pain-PSQI Moderation Analysis of ROI-to-ROI Functional Connectivity

In order to investigate how the effect of PSQI on rsFC varies by group, we performed a moderation analysis [50] on the R2R rsFC. That is, we fitted a second-level GLM to each R2R rsFC as the dependent variable. In Wilkinson's notation, the full model was rsFC∼1 + Age + Sex + PAIN_GROUP^*∗*^PSQI. Given the small sample size compared to the large number of biopsychosocial variables that may influence these complex relationships, only variables that either significantly differed between the groups or that are known to significantly impact resting state functional connectivity like sex and age [51–55] were included as covariates. We were mainly interested in evaluating the significance of the PAIN_GROUP × PSQI interaction (PAIN_GROUP: PSQI term in Wilkinson's notation). However, in order to compare with previous studies with older adults [56–58], we also evaluated the main effect of PSQI in the full model and in a simple model: rsFC∼1 + Age + Sex + PSQI. The significance of this contrast was corrected for multiple comparisons by controlling the False Discovery Rate (FDR; *q* < 0.05). Given that the number of connections was large, potentially reducing the discovery of true positives, we also applied the threshold-free network-based-statistics (TFNBS) method [59], which combines network-based-statistics [60] and threshold-free cluster enhancement [61]. For each tested contrast, supra-threshold “uncorrected” networks, namely, *components*, were detected by thresholding their *t*-statistics at a given value *T*. The value *M*(*T*)=*e*(*T*)^0.5^ × *T*^2^ , where *e*(*T*) is the size of the component, was then assigned to each supra-threshold connection. A matrix of *TFNBS scores* for all connections was created by numerically integrating *M*(*T*) across all values of *T* at a step of *dT*=0.1. We then calculated Family-Wise-Error (FWE) corrected *p*-values by comparing the TFNBS score of a connection to the distribution of maximum TFNBS scores across the matrix under the null hypothesis. This null distribution was generated with 1,000 permutations of the original data among participants.

#### 2.3.2. Hypothesis-Based Restriction of the ROIs Used in the Analysis

To avoid an excessive number of tests, which would be detrimental to the sensitivity of detection of true positives, we restricted the ROIs used in the R2R analysis. For this, we defined sleep *ROIs*, i.e., (those overlapping with areas systematically reported in at least two publications) to be structurally and functionally affected by sleep problems and disorders [10, 11, 13–19, 57]. In addition, we created a set of *pain ROIs* (i.e., those affected by chronic pain or activated by pain) as follows: ROIs containing at least one of the coordinates identified by Flodin and colleagues (2016) [25] (see Supplementary Table S2 of their paper) in a meta-analysis of 314 studies indexed by the searched term “pain” in the online neurosynth tool (https://www.neurosynth.org/). We then used the sleep ROIs as *seeds* and the union of sleep and pain ROIs as *targets* (configuration S-SP). In a less restricted ROI configuration, we used the union of sleep and pain ROIs as seeds and targets (SP-SP). In a third configuration, we used the sleep ROIs as seeds and all 132 ROIs of the atlas, covering the whole brain as targets (S-A). In the final configuration, we used the union of the sleep and pain ROIs as seeds and all 132 ROIs of the atlas as targets (SP-A). The use of different ROI configurations allows hypothesis testing at different levels of sensitivity and specificity, which is appropriate for exploratory research. The sleep and pain ROIs are presented in Table 2.

## 3. Results

A total of 186 individuals were screened via telephone, and 142 met the inclusion criteria and thus, participated in the baseline visit. The present study sample includes only older adults with valid resting-state functional MRI data that also completed the PSQI questionnaire (*n* = 48 participants, 46 right-handed and 2 left-handed). The majority of our pain group sample reported the worst pain locations, mainly of musculoskeletal origin located in the knee and back (76%). A minority reported other pain locations, including the arms and hands and neck and shoulders (23.52%). This is consistent with epidemiological studies where older individuals report back pain and osteoarthritis as the two most common musculoskeletal conditions [62,63].

Detailed demographic and clinical characteristics of our community-dwelling older adults are presented in Table 1 and have been previously reported in other studies [36, 65]. There were no significant differences regarding demographic and clinical characteristics between participants with and without pain, except in pain ratings at the time of the MRI and in PSQI scores. There were no significant correlations between PSQI and either the average (*r* = −0.04, *p*=0.79) or the maximum (*r* = −0.1, *p*=0.49) average of the six motion parameters during the resting state neuroimaging, suggesting poorer sleep did not confound our analysis (e.g., individuals with worse sleep did not fall sleep in the scanner).

**Figure 1:**
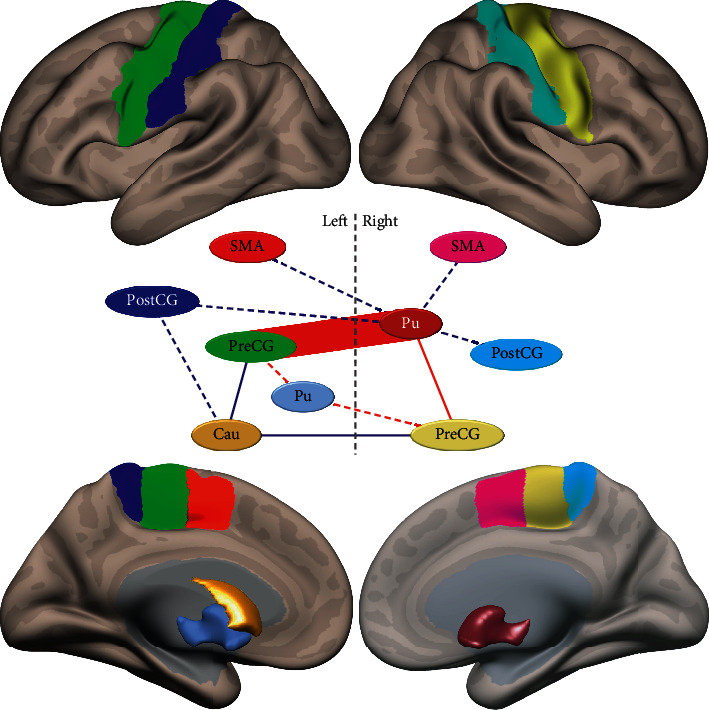
Significant networks in the chronic pain-connectivity moderation analysis of the R2R rsFC when testing the PAIN_GROUP × PSQI interaction. Connections are represented by lines. The PAIN_GROUP × PSQI interaction was negative in all connections. The network formed by all eleven connections survived the TFNBS for the S-SP ROI configuration. The network formed by the blue solid and red connections survived the TFNBS for the SP-SP ROI configuration. The network formed by the red connections survived the TFNBS for the SP-A ROI configuration. The network formed by the solid red lines survived the TFNBS (marginally: left-tailed) for the S-A ROI configuration. The individual connection represented by the thicker red line was significant for all ROI configurations (*p* < 0.05, FDR corrected). The nodes of these networks are ROIs of the Harvard-Oxford AAL atlas. To clarify their anatomical extent, cortical ROIs are shown projected onto a semi-inflated white matter surface and subcortical ROIs are represented in the medial view of this surface. Pu = putamen. Cau = caudate. SMA = juxtapositional lobule cortex. PreCG = precentral gyrus. PostCG = postcentral gyrus.

We found no significant main effect of PSQI with the full or simple models for any ROI configuration. When testing the PAIN_GROUP x PSQI interaction with the S-SP ROI configuration (50 × 63/2 = 1,575 tests), we detected a significant connection between the right putamen and the left precentral gyrus (*p*=0.027, FDR-corrected, two-tailed) and the significant TFNBS network (TFNBS score = 86.8, *p*=0.011, FWE-corrected, two-tailed) shown in Figure 1. This network was formed by eleven connections between the bilateral putamen, left caudate, bilateral precentral gyrus, bilateral postcentral gyrus, and bilateral juxtapositional lobule cortex. With the SP-SP ROI configuration (62 × 63/2 = 1,953 tests), the connection between the right putamen and the left precentral gyrus remained significant (*p*=0.028, FDR-corrected, two-tailed). However, the significant TFNBS network (TFNBS score = 80.6, *p*=0.016, FWE-corrected, two-tailed) reduced to the six connections of the bilateral putamen and left caudate with bilateral precentral gyrus (see Figure 1). With the S-A ROI configuration (62 × 132/2 = 3,300 tests), the connection between the right putamen and the left precentral gyrus was *marginally* significant (*p*=0.038, FDR-corrected, *left-tailed*) and a significant TFNBS network (TFNBS score = 81.4, *p*=0.044, FWE-corrected, two-tailed) was detected, comprising the two connections between the right putamen and bilateral precentral gyrus (see Figure 1). Finally, with the less restricted SP-A ROI configuration (62 × 132/2 = 4,092 tests), the connection between the right putamen and the left precentral gyrus was marginally significant (*p*=0.045, FDR-corrected, left-tailed) and a marginally significant TFNBS network (TFNBS score = 74.4, *p*=0.035, FWE-corrected, left-tailed) was detected, comprising the four connections between bilateral putamen and bilateral precentral gyrus (see Figure 1). As expected, the higher the number of simultaneous tests, the smaller the size and significance of the detected networks. However, two connections were common to the TFNBS networks detected with all ROI configurations: those of the right putamen with bilateral precentral gyrus.

**Figure 2:**
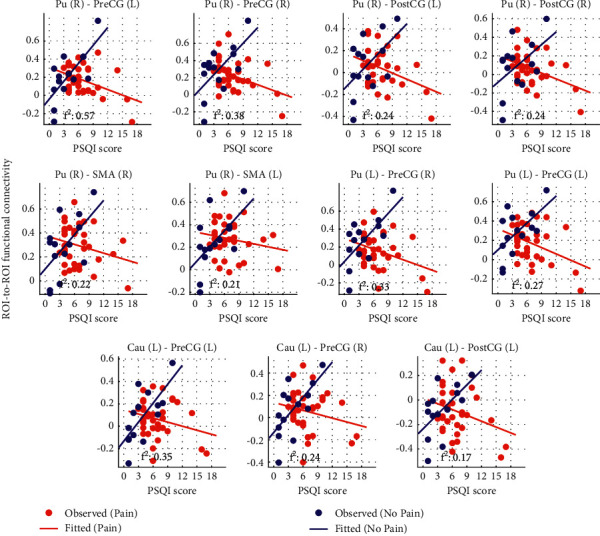
Scatter plot of the rsFC values of the eleven connections that survived the TFNBS in the R2R connectivity analyses (when testing the PAIN_GROUP × PSQI interaction with the S-S ROI configuration) versus PSQI. The values of functional connectivity were adjusted by removing the demeaned residuals explained by age and sex. Thus, for each group (i.e., no-pain and chronic pain) the adjusted fitted rsFC lie in a straight line that represents the slope of the rsFC-PSQI dependency within the group. The negative PAIN_GROUP × PSQI interaction is explained by a change in slope from positive to negative when switching from the no-pain to the chronic pain group. For each connection, the Cohen's *f*^2^ local effect size index is shown, that is, the proportion of variance explained by the PAIN_GROUP × PSQI interaction divided by the residual variance. All effects were medium (0.15 ≤ *f*^2^ < 0.35) or large (*f*^2^ ≥ 0.35) [65]. Pu = putamen. Cau = caudate. SMA = juxtapositional lobule cortex. PreCG = precentral gyrus. PostCG = postcentral gyrus. *R* = right. *L* = left.

The PAIN_GROUP × PSQI interaction was negative for all connections.  Figure 2 depicts, for each group, the relation between PSQI and the R2R rsFC. Greater PSQI scores were associated with greater rsFC in the no-pain group, while this effect was reversed for the chronic pain group, where greater PSQI scores were associated with lower rsFC in those reporting chronic pain. For each of these connections, the Cohen's *f*^2^ local effect size index associated with PAIN_GROUP × PSQI interaction (i.e., the proportion of variance explained by this variable divided by the residual variance [65]) is shown in this figure. Their values indicate that all observed effects were medium to large [65].

## 4. Discussion

The present study aimed to investigate the association between sleep quality, chronic musculoskeletal pain, and rsFC in cognitively intact community-dwelling older adults. Consistent with some previous studies [56, 58] (though see Amorim et al. [57]), we found no significant association between sleep quality and rsFC. However, and as we hypothesized, the association of sleep quality with rsFC was significantly dependent on the presence of chronic musculoskeletal pain. This moderation occurred in a network in which the dorsal striatum (i.e., bilateral putamen and left caudate) was connected to the motor and somatosensory cortices. For all ROI configurations, the connections between the right putamen and bilateral precentral gyrus survived TFNBS significance, and the connection between the right putamen and the left precentral gyrus was individually significant (*p* < 0.05, FDR-corrected). Our results suggest that, for older adults with no reported chronic musculoskeletal pain, greater rsFC within this network is associated with poorer sleep quality. Conversely, rsFC seems to decrease with poorer sleep quality when chronic musculoskeletal pain is present.

The association of worse sleep quality with greater rsFC of the dorsal striatum and sensorimotor cortices in the control group appears to contradict the findings reported by Curtis et al. [17], where greater sleep duration in hours was associated with less rsFC between these same areas. However, that study did not account for the widespread age of the sample, spanning from 18 to 64 years. An increase in self-reported sleep problems with age was previously reported by others using the PSQI score [66–68]; and rsFC was observed to decrease with age within sensorimotor networks [52, 53, 69–72] and between sensorimotor areas and the putamen [73]. Thus, we cannot discard the possibility that the effects observed by Curtis et al. [17] could be age-related, and thus, their results might not contradict ours. Another study [11], with results more in agreement with ours, reported greater rsFC between the right putamen and the left ventral premotor cortex in chronic primary insomnia patients compared to healthy controls, where a wide age range (25–65 years) was controlled for. However, we cannot directly compare the results from an insomnia sample with those from our generally healthy individuals without diagnosed insomnia. In general, there is a paucity of studies in the literature, even in younger samples, showing an association between sleep quality and dorsostriatal-sensorimotor rsFC in healthy individuals. This renders a difficult comparison of our results with the literature, a reason that propels the need for future studies examining sleep quality and rsFC across the lifespan while also considering pain status.

An association between dorsostriatal functional networks and sleep quality is strongly supported by accumulated evidence in favor of a fundamental role of the basal ganglia in regulating the sleep-wake cycle [74]. The dorsal striatum modulates behavior [75–77] via connections with sensorimotor and association cortices [78, 79] and is believed to enhance wakefulness [74]. Thus, it is possible that the greater connectivity in dorsostriatal-sensorimotor associations with worse sleep quality in the control group reflects greater corticostriatal coupling needed to maintain a given level of wakefulness in poorer sleepers.

This positive correlation between the dorsostriatal-sensorimotor connectivity and PSQI in the control group could also be related to an increased state of somatomotor hyperarousal caused by poorer sleep quality, resembling what has been observed in insomnia [80]. The dorsal striatum is part of the motor circuit [74, 78, 79, 81, 82]. The putamen has a role in motor learning and control of initiated movements [81–83], especially of body and eye movements [84], and the caudate transforms spatial information (via working memory) into motor behavior, controlling and inhibiting body and limbs posture and speed [75, 85, 86]. Thus, the increase in dorsostriatal-sensorimotor rsFC could reflect mild “motor restlessness” associated with poorer sleep quality. Motor restlessness, characterized by an irresistible urge to move, is believed to be a manifestation of physiological arousal associated with many underlying disorders [87]. This line of reasoning is consistent with reports that motor restlessness and similar syndromes were associated with significantly worse sleep quality [88].

Conversely, dorsostriatal-sensorimotor rsFC decreased with worse sleep quality in those reporting chronic pain. This suggests that the abovementioned mechanisms by which dorsostriatal-sensorimotor networks are increasingly recruited with poorer sleep quality in no-pain older adults are disrupted in those with chronic pain. This might be due to a shift in the recruitment of the dorsal striatum toward its consistently reported and pivotal regulatory function of sensorimotor features of the pain experience [89, 90] (e.g., caudate modulation of the motor response associated with pain avoidance [91]). This shift may be compensatory and/or aberrant related to structural and functional alterations of the striatum associated with chronic pain. For example, a continuous drive of activity associated with chronic musculoskeletal pain may lead to abnormal increases in putaminal volume [92, 93], leading to sensory deficits, as infarction data suggests [94]. Chronic pain-related structural striatal alterations, in tandem with related abnormal inputs from peripheral systems and cortical and subcortical regions, could lead to altered striatal functions and impaired cortico-striatal loops [90]. This would be consistent with our diminished dorsostriatal-sensorimotor functional connectivity in our chronic pain group. Future studies are needed to replicate these findings and to further understand the neurobiological mechanisms underlying the negative relation between PSQI and rsFC observed in the chronic pain group.

The present study has some limitations. Our sample is small, limiting the statistical power to detect small effects. Second, functional MRI connectivity offers no information about the directionality of connections, limiting the mechanistic interpretation of results. Also, our results could be specific to older adults and cannot be generalized to younger or middle-aged individuals. Moreover, our findings cannot be generalized to individuals with severe and diagnosed sleep problems, as these were excluded in the present study. It is also worth mentioning that, although self-reported pain is the gold standard to define participants with chronic musculoskeletal pain [30, 31, 33], other measures, like quantitative sensory testing (QST), could be explored in the future. In summary, studies with larger sample sizes, wider age ranges, including more severe sleep problems and more objective pain measures, may provide further insight into the mechanisms underlying the interplay between chronic pain and sleep quality across the lifespan.

## 5. Conclusions

Our study provides initial evidence that the presence of chronic musculoskeletal pain in older adults impacts how sleep quality correlates with resting-state functional connectivity in striatal-sensorimotor loops. Our results suggest that while the state of hyperarousal increases with poorer sleep quality in the no-pain group, this is reversed in the chronic pain group, possibly owing to the recruitment of the striatum toward regulatory sensorimotor functions related to the pain experience or impaired corticostriatal-thalamic loops associated with chronic pain. In this study, we shed light on the neurobiological mechanisms underlying sleep problems in older adults and their associations with chronic musculoskeletal pain, prevalent comorbidity in this vulnerable group. We expect that understanding the neurobiological underpinnings of pain and sleep in older adults can help pave the road to developing additional therapeutic targets in our increasingly aging population.

## Figures and Tables

**Table 1:** Demographics and clinical characteristic of the sample (*n* = 48).

	Chronic pain (*n* = 34)	No chronic pain (*n* = 14)	Significance
Age, mean ± SD	72 ± 6.78	74.50 ± 7.31	0.283 (*t*-test)
Sex, no. (%)			0.072 (*χ*^2^)
Male	8 (23.53%)	7 (50%)	
Female	26 (76.47%)	7 (50%)	
Race, no. (%)			0.651 (*χ*^2^)
Caucasian	32 (94.12%)	14 (100%)	
Other	2 (5.88%)	0 (0%)	
Education level, no. %			0.067 (*χ*^2^)
High school	10 (29.41%)	3 (21.43%)	
Two year college	7 (20.59%)	0 (0%)	
Four year college	7 (20.59%)	1 (7.14%)	
Master's degree	7 (20.59%)	7 (50%)	
Doctorate's	3 (8.82%)	2 (14.29%)	
		1 missing data	
Marital status, no. %			0.559 (*χ*^2^)
Married	17 (50%)	7 (50%)	
Other	17 (50%)	5 (35.71%)	
		2 missing data	
Income, no. %			0.076 (*χ*^2^)
Less than $15,000	3 (8.82%)	0 (0%)	
$15,000 to $25,000	5 (14.71%)	1 (7.14%)	
$25,000 to $40,000	5 (14.71%)	0 (0%)	
$40,000 to $55,000	8 (23.53%)	1 (7.14%)	
$55,000 to $70,000	1 (2.94%)	3 (21.43%)	
Higher than $70,000	12 (35.29%)	6 (42.86%)	
		3 missing data	
CES-D, mean ± SD	7.26 ± 5.18	5.64 ± 4.65	0.298 (*t*-test)
PANAS positive	34.21 ± 9.66	35.82 ± 6.11	0.525 (*t*-test)
PANAS negative	11 ± 1.71	11 ± 1.79	1.00 (*t*-test)
STAI-trait	28.67 ± 4.66	26.82 ± 3.82	0.204 (*t*-test)
STAI-state	26.24 ± 7.92	25.55 ± 4.91	0.733 (*t*-test)
MoCA, mean ± SD	26.03 ± 2.61	27.29 ± 2.09	0.090 (*t*-test)
Total PSQI, mean ± SD	6.88 ± 3.46	3.93 ± 3	**0.006** ^*∗*^ (*t*-test)
Momentary pain intensity at MRI (0–100 scale), mean ± SD	12.64 ± 15.20	1.14 ± 2.88	**0.008** ^*∗*^ (*t*-test)

*Note*. ^*∗*^significant *p*-value after Bonferroni correction (i.e., *p*=0.001).

**Table 2:** List of the seeds ROIs used in the R2R connectivity analysis.

Description	Sleep		Pain	
	Left	Right	Left	Right
Frontal pole	[10, 16]	[10, 12, 16]	—	[25]
Insular cortex	[13, 15]	[10, 11, 13, 15, 18, 57]	[25]	[25]
Superior frontal gyrus	—	[12, 16]	—	—
Middle frontal gyrus	[10, 11]	[10, 11]	—	[25]
Inferior frontal gyrus, par triangularis	—	—	—	[25]
Inferior frontal gyrus, par opercularis	—	—	[25]	[25]
Precentral gyrus	[10, 17]	[10, 15, 17]	[25]	[25]
Temporal pole	[14, 15]	[14, 15, 57]	—	—
Middle temporal gyrus, posterior division	[16, 57]	—	—	—
Middle temporal gyrus, temporooccipital part	[15, 57]	—	—	—
Postcentral gyrus	[10, 11, 15–17]	[10, 11, 15, 17]	[25]	[25]
Superior parietal lobule	—	—	[25]	—
Supramarginal gyrus, anterior division	[10, 57]	[10, 11, 16, 57]	[25]	[25]
Supramarginal gyrus, posterior division	[10, 16, 57]	[10, 16, 57]	—	[25]
Angular gyrus	[14, 57]	[14, 57]	—	—
Lateral occipital cortex, inferior division	[10, 16, 17]	[10, 15–17]	—	—
Intracalcarine cortex	—	[11, 57]	—	—
Frontal medial cortex	[10, 16, 57]	[25]		
Juxtapositional lobule cortex	[17, 18]	[17, 18]	[25]	[25]
Paracingulate gyrus	[10, 18, 19]	[10, 18, 19]	[25]	[25]
Cingulate gyrus, anterior division	[11, 13]	[25]		
Cingulate gyrus, posterior division	[10, 11, 18, 57]	[25]		
Precuneous	[10–12, 19, 57]	—	—	
Frontal orbital cortex	[11, 57]	[10, 11]	—	[25]
Occipital fusiform gyrus	[16, 57]	—	—	—
Frontal operculum cortex	—	[11, 15, 57]	[25]	[25]
Central operculum cortex	—	—	[25]	[25]
Parietal operculum cortex	[10, 57]	[10, 57]	[25]	[25]
Heschl's gyrus	[15, 17, 57]	—	—	[25]
Planum temporale	[10, 57]	[10, 57]	—	—
Thalamus	—	—	[25]	[25]
Caudate	[15–17, 57]	[11, 12, 15–17, 57]	—	[25]
Putamen	[15, 17]	[10, 11, 15–17]	[25]	[25]
Pallidum	—	—	—	[25]
Hippocampus	[10, 12, 17, 18, 57]	[10, 11, 57]	—	[25]
Amygdala	[10, 15, 57]	[10, 15, 57]	[25]	[25]
Accumbens	[16, 57]	—	—	—
Brain-stem	—	[25]		
Cerebelum crus I	[17, 57]	—	—	—

*Note*. ROIs containing brain areas systematically reported to be structurally and/or functionally affected by sleep disorders (sleep ROIs) and/or affected or activated by pain (pain ROIs). Each condition (i.e., sleep or pain) has two columns corresponding to the left and right hemispheres. However, some ROIs located around the middle plane (e.g., the Precuneus) cover both hemispheres and the columns were merged. For each condition and hemisphere, publications systematically reporting a structure within an ROI (at least two publications for a sleep ROI) are referenced.

## Data Availability

Data are available upon request.
